# Fabrication of water-soluble polymer-encapsulated As_4_S_4_ to increase oral bioavailability and chemotherapeutic efficacy in AML mice

**DOI:** 10.1038/srep29348

**Published:** 2016-07-07

**Authors:** Qiang Ma, Chuan Wang, Xiaojin Li, Hua Guo, Jie Meng, Jian Liu, Haiyan Xu

**Affiliations:** 1 Institute of Basic Medical Sciences Chinese Academy of Medical Sciences, School of Basic Medicine Peking Union Medical College, Beijing 100005, P. R. China

## Abstract

Realgar (As_4_S_4_) has been demonstrated to be effective for the treatment of acute myeloid leukemia (AML); it has the advantages of no drug resistance and oral administration. Nevertheless, its poor solubility has been an obstacle to its bioavailability, requiring high-dose administration over a long period. We investigated whether crushing realgar crystals to the nanoscale and encapsulating the particles in a water-soluble polymer in one step using hot-melt extrusion would increase the bioavailability of As_4_S_4_. Raw As_4_S_4_ (r-As_4_S_4_) and water-soluble polymer were processed via co-rotating twin screw extrusion. The resulting product (e-As_4_S_4_) was characterized by SEM, XRD, and DLS. The cytotoxicity and therapeutic effects of e-As_4_S_4_ were evaluated *in vivo* and *in vitro*. The results show that e-As_4_S_4_ dissolved rapidly in water, forming a stable colloid solution. The average size of e-As_4_S_4_ particles was 680 nm, which was reduced by more than 40-fold compared with that of r-As_4_S_4_. The bioavailability of e-As_4_S_4_ was up to 12.6-fold higher than that of r-As_4_S_4_, and it inhibited the proliferation of HL-60 cells much more effectively than did r-As_4_S_4_, inducing apoptosis and significantly reducing the infiltration of HL-60 cells into the bone marrow, spleen, and liver. This in turn prolonged the survival of AML mice.

Acute myeloid leukemia (AML) is the most common acute leukemia, making up 70% of cases^1^. Although the first remission in AML patients can generally be reached, at a rate of 50–85%, by the standard treatment of 7 days of standard-dose cytarabine (Ara-C) following 3 days of anthracycline, the survival rate of AML patients is still the lowest of all leukemias^2^, because relapse occurs in 50–70% of remission patients within 3 years. This is accompanied by drug resistance^3^, representing a major obstacle to any cure. Thus, it is of great significance to develop alternative treatments for patients with relapse and those who fail to enter complete remission.

Arsenic trioxide (ATO) induces complete remission (CR) in more than 80% of refractory patients and those with relapse^4^,^5^. Furthermore, high rates of CR and 5-year disease-free survival (DFS) above 90% can be achieved with a combination of ATRA and ATO during induction followed by their sequential application during maintenance therapy^6^,^7^,^8^,^9^. However, ATO must be administrated intravenously daily in a 1–4 h infusion, and it has notable toxic effects^10^.

Realgar, crystallized As_4_S_4_, is a mineral drug that is in wide use in traditional Chinese medicine (TCM). Early clinical exploration of As_4_S_4_ for the treatment of AML and CML started in the 1960s^11^, and suggested that As_4_S_4_ was a potent agent for leukemia treatment. Subsequent studies have provided more evidence that several oral formulae containing As_4_S_4_ have therapeutic effects on leukemia^12^,^13^,^14^,^15^,^16^,^17^,^18^,^19^. For example, CR rates of 98%^14^ and 96.7%^18^ were reached for acute promyelocytic leukemia (APL) patients orally administered realgar–indigo naturalis formula (RIF), and a 5-year overall survival (OS) rate of 86.9% was achieved^19^. Furthermore, a clinical trial reported that As_4_S_4_ alone was effective and safe for both remission induction and maintenance therapy in patients with APL, regardless of disease stage^10^. The other components of RIF promote the transport of arsenic into cells by enhancing aquaglyceroporin 9 expression^20^. A multicenter, randomized, controlled trial reported that oral RIF plus ATRA was not inferior to intravenous ATO plus ATRA as induction and maintenance therapies for newly diagnosed acute APL and may be considered a routine treatment option in appropriate patients^15^. Recently, a single-center pilot study demonstrated that RIF treatment was effective and convenient for treating low-risk APL patients^17^. Based on these clinical achievements, RIF has been approved by the China Food and Drug Administration (CFDA)^18^ and recommended as a second-line chemotherapeutic agent for the treatment of APL in China^10^,^21^.

Nevertheless, a major drawback of As_4_S_4_ is that only 0.6% of the total arsenic content is leachable under simulated alimentary tract conditions^22^ because it is poorly soluble in neutral and acidic conditions due to its crystal structure. The bioavailability of As_4_S_4_ in the formula of Niu Huang Jie Du Pian was reported to be only 4%^23^. Thus, patients have to be administered As_4_S_4_ at high doses for up to several years to obtain effective concentrations of arsenic in the blood, which can cause side effects such as asymptomatic prolongation of corrected QT interval, transient elevation in liver enzyme levels, rash, and mild gastrointestinal discomfort^10^, and can cause a heavy burden in terms of medical care as well. Some strategies for enhancing the bioavailability of As_4_S_4_ have been developed, such as preparing nanosized realgar particles by cryo-grinding with polyvinyl pyrrolidone (PVP) and/or sodium dodecyl sulfate (SDS)^24^, using ball-milling and solvent-evaporation techniques to prepare realgar-based microcapsules with gelatin^25^, and directly reducing particle size to the nanoscale via high-energy ball milling^26^,^27^. Another approach is to react realgar with ethylene amine to form As_4_S_4_ nanoclusters and further to form a nanogel by introducing carboxylic acid into the synthesis system^28^.

Hot-melt extrusion (HME) is one of the most widely used processing technologies in chemical and materials sciences to develop materials with a certain performance, such as composites containing carbon nanotubes^29^,^30^, graphene^31^, and layered silicate^32^, and also fiber-enhanced polymer materials^33^. Today, this technology has also found its place in the pharmaceutical field for the manufacture of various doses and formulations of drugs^34^,^35^,^36^, suggesting that the technology is effective for improving the dissolution rate of organic drugs with poor water solubility. However, solid dispersion formulations for inorganic mineral drugs using HME have rarely been reported. To overcome the high intrinsic lattice energy of As_4_S_4_ crystal particles, we used HME to co-extrude raw As_4_S_4_ (r-As_4_S_4_) with one kind of water-soluble polymer to fabricate a novel solid dispersion formulation (e-As_4_S_4_). We showed that in the co-extrusion, r-As_4_S_4_ was crushed into nanoscale particles and encapsulated by the water-soluble polymer. The resulting product e-As_4_S_4_ dissolved rapidly in saline and simulated gastric and intestinal juices, increasing the bioavailability of r-As_4_S_4_ significantly. Indeed, in tests on mice with AML, compared to r-As_4_S_4,_ e-As_4_S_4_ had much stronger inhibitory effects on the proliferation of HL-60 cells and significantly prolonged the survival of the mice.

## Results

### Co-extrusion changes the particle size and crystalline structure of r-As_4_S_4_

**Figure 1:**
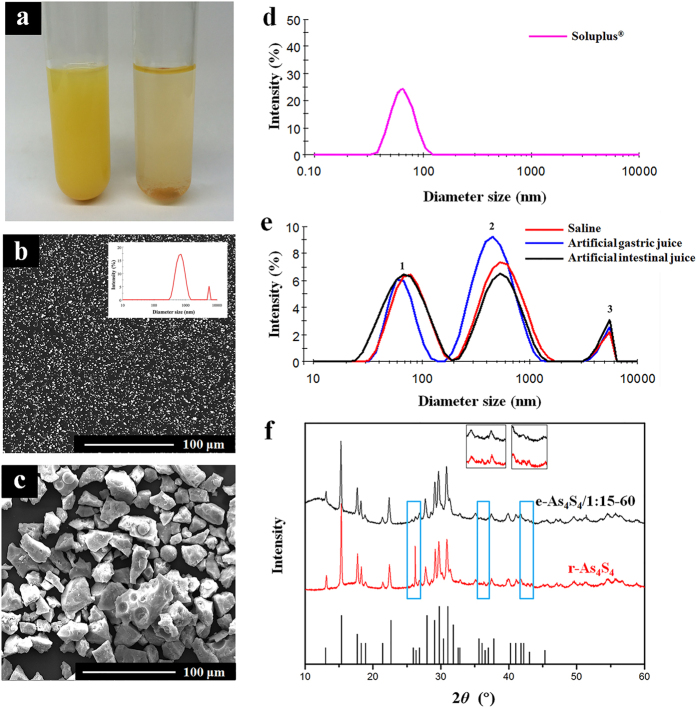
Morphology and solubility studies of e-As_4_S_4_ and r-As_4_S_4_. (**a**) e-As_4_S_4_/1:15–60 could be dissolved in saline (left: e-As_4_S_4_, right: r-As_4_S_4_). (**b**,**c**) SEM images of realgar particles in e-As_4_S_4_/1:15–60 (**b**) and r-As_4_S_4_ (**c**). Scale bar indicates 100 μm. The inserted graph in (**b**) shows the size distribution obtained from DLS measurement. (**d**,**e**) Hydrodynamic diameter distribution of PVCL-PVAc-PEG dissolved in saline (**d**) and e-As_4_S_4_/1:15–60 (**e**) dissolved in different media (saline: red line; artificial gastric juice: blue line; artificial intestinal juice: black line). (**f**) Powder-XRD patterns of e-As_4_S_4_/1:15–60 (top) and r-As_4_S_4_ (middle). The JCPDS card pattern of α-As_4_S_4_ (No. 09–0441) is also shown for comparison.

In the first set of experiments, we investigated and compared the particle size and crystalline structures of r-As_4_S_4_ and e-As_4_S_4_. When added to water with 10 min of sonication, e-As_4_S_4_/1:15–60 dissolved rapidly and formed a yellow colloid solution (Fig. 1a left), whereas r-As_4_S_4_ mainly precipitated out at the bottom of the container (Fig. 1a right). The average diameter of e-As_4_S_4_/1:15–60 particles was about 680 nm, as determined from SEM images (Fig. 1b), significantly smaller than r-As_4_S_4_ particles (28.9 μm; Fig. 1c). The size distributions of e-As_4_S_4_/1:15–60 in different aqueous media were analyzed by DLS. Samples had three peaks in DLS graphs when dissolved in saline, where peak 1 was attributable to micelles formed by polymer molecules alone (Fig. 1d). Peak 2 represented e-As_4_S_4_ encapsulated in PVCL-PVAc-PEG molecules (Fig. 1e), showing a typical Gaussian distribution with an average hydrodynamic diameter of 700 nm, consistent with the average size obtained from SEM. Peak 3 represented a very small proportion of larger particles in the solution. Peak 2 accounted for ~55% of the sum of the three peaks in terms of peak area. Furthermore, e-As_4_S_4_ in artificial gastric juice or artificial intestinal juice showed similar DLS patterns to that in saline, with only slight variation in the proportion of peak 2; the largest proportion was seen in artificial gastric juice (66%) and the lowest was observed in artificial intestinal juice (45%). These results indicate that the particle size of e-As_4_S_4_ was decreased more than 40-fold. In addition to the marked size reduction, the crystal structure of r-As_4_S_4_ changed slightly. Figure 1f shows the XRD patterns of r-As_4_S_4_ and e-As_4_S_4_/1:15–60. All of the diffraction peaks of r-As_4_S_4_ could be indexed to those filed on JCPDS card file (No. 09–0441) for the α-As_4_S_4_ crystal. However, several peaks in the pattern of e-As_4_S_4_ showed decreased intensity or even disappeared. A higher background was observed in the range of 10–20° with reference to that in the pattern of r-As_4_S_4_, suggesting the presence of amorphous As_4_S_4_, possibly because some part of the crystal structure was destroyed during the extrusion process.

### Dissolution rate and bioavailability are higher in e-As_4_S_4_ *in vivo*

**Figure 2:**
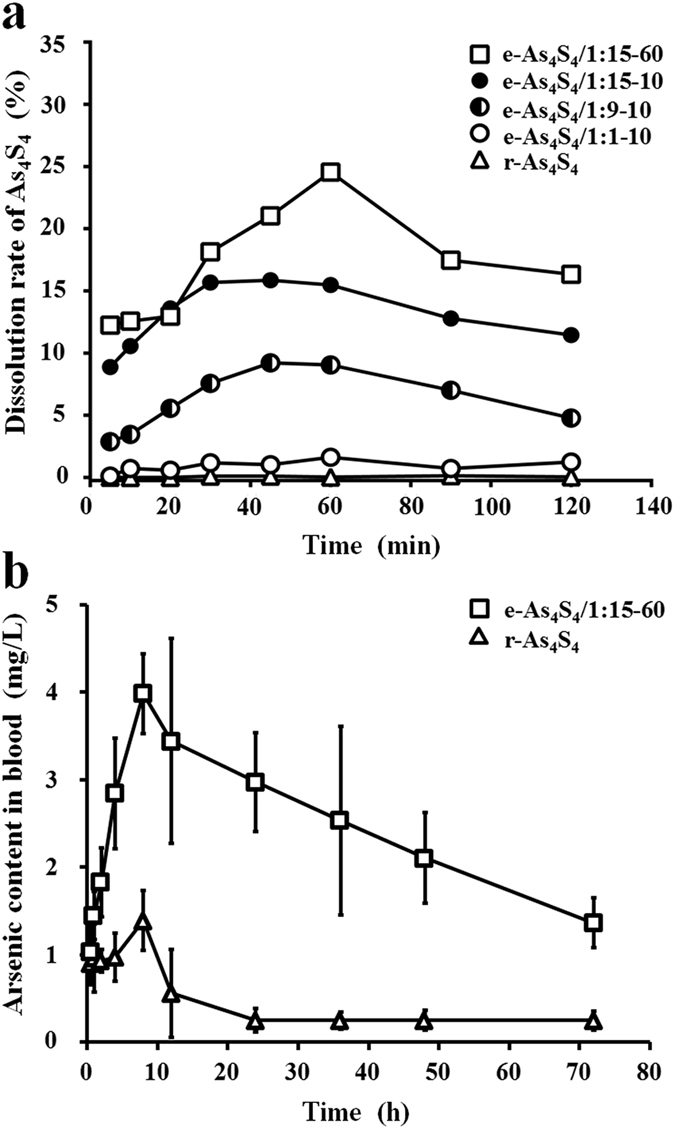
Dissolution behavior and oral bioavailability of As_4_S_4_ in different formulations. (**a**) Dissolution rates of As_4_S_4_ in e-As_4_S_4_/1:15–60, e-As_4_S_4_/1:15–10, e-As_4_S_4_/1:9–10, e-As_4_S_4_/1:1–10, and r-As_4_S_4_ in 0.1 M HCl at 37 ± 0.5 °C with gentle mechanical stirring at 100 rpm (according to the guidance of the CHP 2010 Apparatus III). (**b**) Bioavailability of As_4_S_4_ in e-As_4_S_4_/1:15–60 and r-As_4_S_4_ after oral administration in rats.

Dissolution is a key factor affecting the bioactivity of drugs. Thus, we examined whether e-As_4_S_4_ showed improved dissolution and bioavailability. The dissolution rates of e-As_4_S_4_ and r-As_4_S_4_ were determined by ICP-OES (Fig. 2a). In 0.1 M HCl, little r-As_4_S_4_ dissolved; the highest dissolution rate was only 0.12%. Strikingly, e-As_4_S_4_, in particular e-As_4_S_4_/1:15–60, had a much higher dissolution rate, largely dissolving within the first 45 min. The dissolution rate increased over time, reaching its highest value of 24.55% after 60 min of immersion.

Next, the bioavailabilities of e-As_4_S_4_/1:15–60 and r-As_4_S_4_ were examined and compared in SD rats treated using intragastric administration. The concentration of arsenic in blood was determined at designated time points after intragastric administration (Fig. 2b). After 0.5 h of administration, arsenic was detectable in the blood of all rats, indicating that the animals had absorbed the compound. In the r-As_4_S_4_ group, the plasma concentration of arsenic increased within the first 8 h, reaching a peak value of 1.3 ± 0.3 mg/L at 8 h, and then decreased quickly to background levels within 24 h after administration. A marked difference was observed in rats given e-As_4_S_4_, namely, the plasma concentration of arsenic increased much faster. Moreover, a much higher peak value was detected, at 3.7 ± 0.5 mg/L at 8 h after administration, and the effects were markedly longer lasting. Even at 72 h after administration, the concentration of arsenic in plasma was still 1.0 ± 0.2 mg/L, approximately equal to the highest level achieved in the r-As_4_S_4_ group. The bioavailabilities of e-As_4_S_4_ and r-As_4_S_4_ (area under the curve, AUC) were calculated (Table 1), indicating that the bioavailability of e-As_4_S_4_ was 12.6 times that of r-As_4_S_4_.

### e-As_4_S_4_ has strong inhibitory effects on the growth of HL-60 cells and induces apoptosis

**Figure 3:**
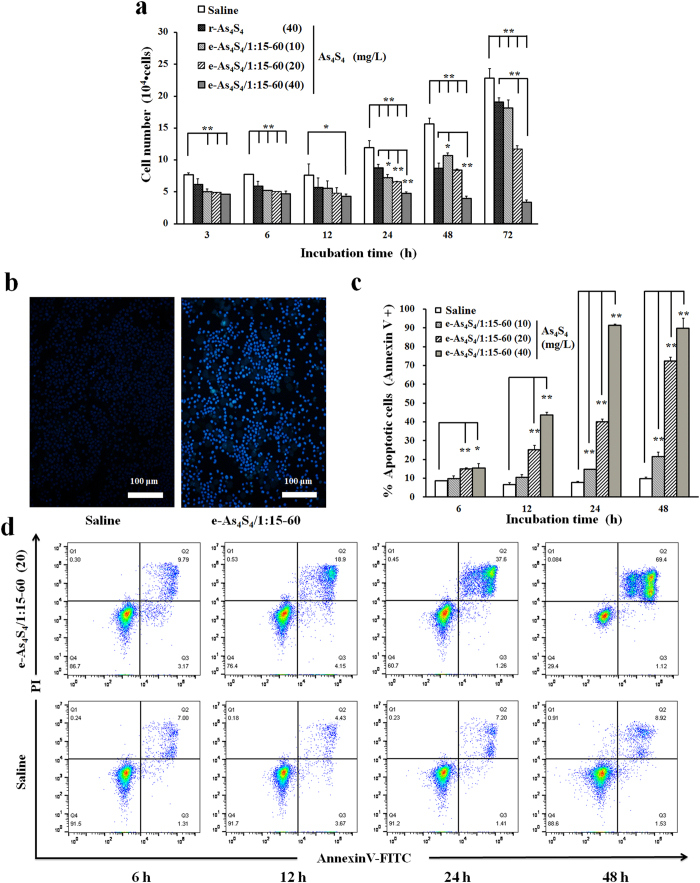
Cytotoxicity of e-As_4_S_4_ and r-As_4_S_4_ toward HL-60 cells. (**a**) The number of viable cells was associated with As_4_S_4_ concentration and incubation times of r-As_4_S_4_ and e-As_4_S_4_/1:15–60 (*n* = 4). **P < 0.01, *P < 0.05. (**b**) Apoptosis of HL-60 cells incubated with e-As_4_S_4_/1:15–60 at 37 °C for 48 h. The HL-60 cells were cultured in medium containing 40 mg/L As_4_S_4_ and stained with Hoechst 33342. The scale bar in all images indicates 100 μm. (**c**) Apoptosis in HL-60 cells was induced by e-As_4_S_4_/1:15–60 in a dose- and time-dependent manner. HL-60 cells incubated with e-As_4_S_4_/1:15–60 containing different amounts of As_4_S_4_ (10–40 mg/L) for 6–48 h were assessed by flow cytometry after annexin V staining. **P < 0.01, *P < 0.05. (**d**) The apoptosis percentage of HL-60 cells incubated with e-As_4_S_4_/1:15–60 20 mg/L As_4_S_4_ for 6–48 h was measured by flow cytometry after annexin V/PI staining.

The cytotoxicities of three formulations were examined (e-As_4_S_4_/1:3–60, e-As_4_S_4_/1:9–60, and e-As_4_S_4_/1:15–60) and the last had the highest cytotoxicity in HL60 cells, although the difference between e-As_4_S_4_/1:15–60 and e-As_4_S_4_/1:9–60 was slight (Supplementary Fig. S1). Because e-As_4_S_4_/1:15–60 also exhibited the best dissolution and dispersion among the three formulations, e-As_4_S_4_/1:15–60 was chosen for the following investigations. In addition, the cytotoxicity of the polymer (PVCL-PVAc-PEG) was evaluated. It was not toxic to cells within 72 h incubation at the arsenic concentrations used in the cell viability assay for e-As_4_S_4_ (Supplementary Fig. S2). To investigate whether e-As_4_S_4_/1:15–60 had stronger cytotoxicity on HL-60 cells than did r-As_4_S_4_, the testing concentration of As_4_S_4_ was set at 10–40 mg/L. At the same As_4_S_4_ content, e-As_4_S_4_ exhibited much higher cytotoxicity than r-As_4_S_4_ at each time point. The cytotoxicity of e-As_4_S_4_ at 10 mg/L As_4_S_4_ was approximately equal to that of r-As_4_S_4_ at As_4_S_4_ 40 mg/L (Fig. 3a). To examine the underlying mechanism of the inhibitory effect, HL-60 cells treated with e-As_4_S_4_/1:15–60 were stained with Hoechst 33342 and subjected to fluorescence microscopy. Strong blue fluorescence was observed in the cells, indicating that e-As_4_S_4_ induced apoptosis (Fig. 3b). Quantification of apoptosis was performed using annexin V/PI staining and flow cytometry. The percentage of apoptosis was dependent on the concentration of e-As_4_S_4_ and the incubation time (Fig. 3c,d). When treated with e-As_4_S_4_ at 10 mg/L As_4_S_4_, apoptosis was not significant until 24 h; however, when treated with e-As_4_S_4_ at 40 mg/L As_4_S_4_, the HL-60 cells underwent apoptosis in the first 6 h of incubation. These results clearly indicate that e-As_4_S_4_ significantly inhibited the growth of HL-60 cells, by inducing apoptosis.

### Oral administration of e-As_4_S_4_ prolongs the survival of AML animals

**Figure 4:**
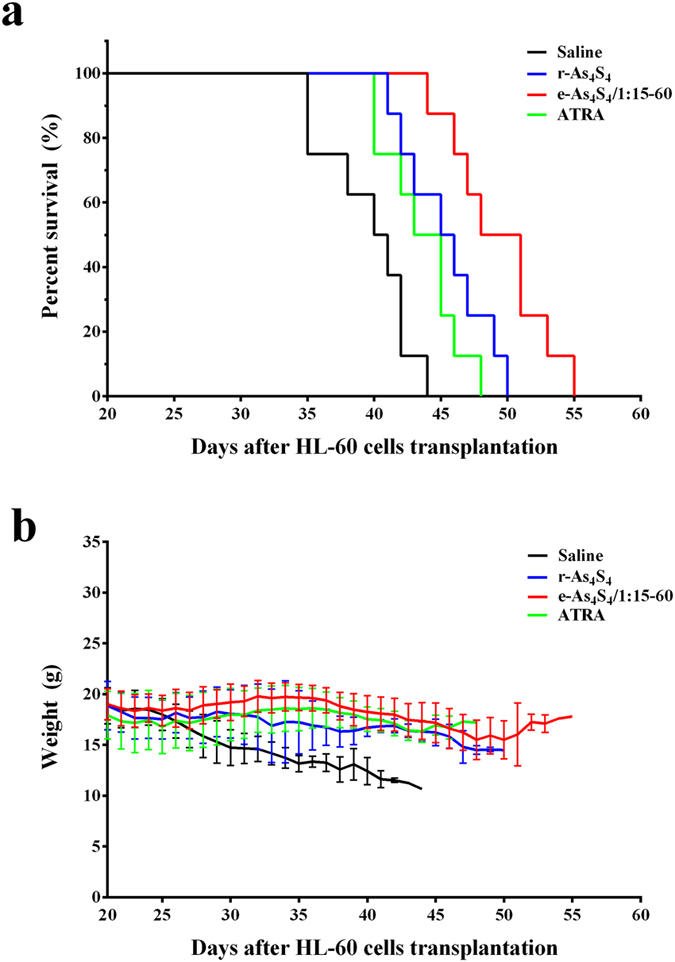
*In vivo* therapeutic efficacies of r-As_4_S_4_, e-As_4_S_4_, and ATRA. (**a**) Survival curve of AML mice treated with saline (blue line), r-As_4_S_4_ (red line), e-As_4_S_4_/1:15–60 (green line), or ATRA (purple line) (*n* = 8). The log-rank test (Mantel-Cox) was used to assess statistical significance: e-As_4_S_4_ vs. saline (*P* < 0.0001), e-As_4_S_4_ vs. r-As_4_S_4_ (*P* = 0.0264), e-As_4_S_4_ vs. ATRA (*P* = 0.0049), r-As_4_S_4_ vs. ATRA (*P* = 0.2082). (**b**) Body weight change curves of AML mice treated with saline (blue line), r-As_4_S_4_ (red line), e-As_4_S_4_/1:15–60 (green line), or ATRA (purple line) (*n* = 8). NOD/SCID mice were injected intravenously with HL-60 cells (1 × 10^6^ cells/mouse) after 250 cGy γ-irradiation. After 20 days, treatment was started, with intragastric administration of saline, r-As_4_S_4_ (1.8 mg per mouse per time, twice per day), e-As_4_S_4_/1:15–60 (28.8 mg per mouse per time, twice per day), or ATRA (0.18 mg per mouse per time, once per day).

On day 18 after HL-60 cell transplantation, mice showed marked leukemic symptoms, including paresis in the rear limbs, ruffled fur, and a markedly hunched posture compared with healthy control mice. On day 20 after transplantation, the leukemic mice were divided randomly into four groups and administered e-As_4_S_4_/1:15–60, r-As_4_S_4_, ATRA, or saline. Although the survival of the mice in each treatment group was prolonged to different degrees versus those in the untreated group, the mice in the e-As_4_S_4_ group showed the longest survival, with statistically significant differences versus the other groups (Fig. 4a). Moreover, during the first 3-week treatment, mice in the e-As_4_S_4_ group did not exhibit significant weight loss, maintaining the best weight level among the four groups (Fig. 4b), indicating that e-As_4_S_4_ was the most effective treatment in the experiment. An abnormal increase in body weight in the very late stage of survival in the e-As_4_S_4_ group was attributable to ascites production.

**Figure 5:**
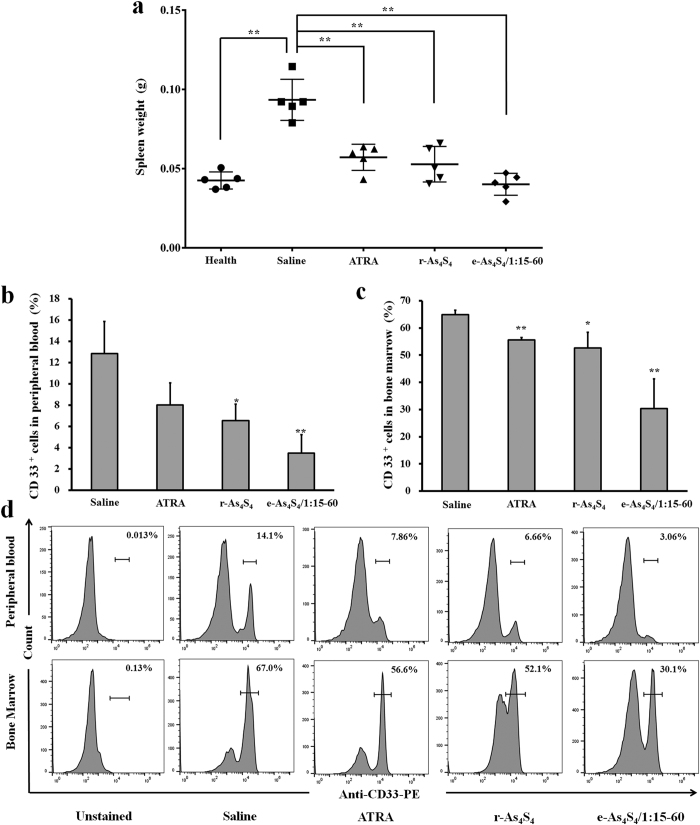
Spleen weight (**a**) and percentage of CD33^+^ cells in peripheral blood (**b**) and in bone marrow (**c**) of mice after 3 weeks treatment with saline (untreated group), r-As_4_S_4_, e-As_4_S_4_, or ATRA (*n* = 5). The CD33^+^ cell percentages in peripheral blood and bone marrow of mice after 3 weeks of treatment were measured by flow cytometry after anti-human CD33 antibody staining (**d**). **P < 0.01 or *P < 0.05, vs. corresponding value in the saline group.

**Figure 6:**
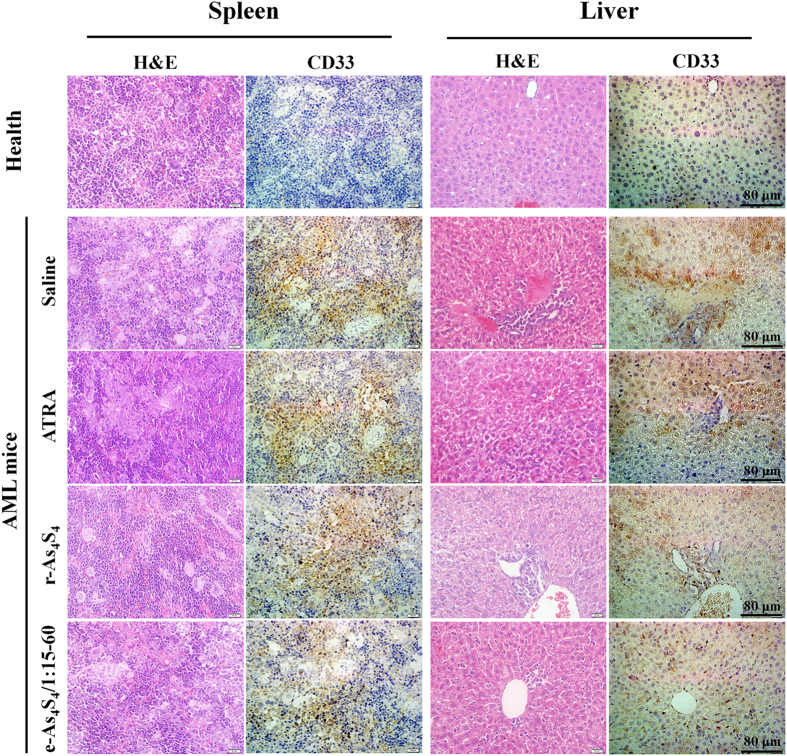
Histological and immunohistochemical analyses of spleens and livers collected from NOD/SCID mice after 3 weeks treatment with saline, ATRA, r-As_4_S_4_, or e-As_4_S_4_. The CD33-positive area was stained brown. Scale bar indicates 80 μm.

Splenomegaly is a typical symptom resulting from leukemia cell infiltration. This condition was relieved to different degrees depending on treatment, evidenced by reduced spleen weight. In particular, the e-As_4_S_4_ group exhibited the lowest average spleen weight, which was similar to that of healthy mice (Fig. 5a). Moreover, the percentage of HL-60 cells (CD33^+^) decreased significantly, from 13% to 3% in peripheral blood and from 65% to 30% in bone marrow, after e-As_4_S_4_ treatment (Fig. 5b,c). Furthermore, H&E staining and anti-CD33 antibody staining were used to detect extramedullary infiltration in the spleen and liver. Mice in the untreated group showed severe diffuse infiltration of HL-60 cells in the spleen and liver, particularly in the splenic sinus and hepatic sinusoid. After receiving e-As_4_S_4_ treatment for 3 weeks, extramedullary infiltration in spleens and livers was clearly reduced (Fig. 6).

## Discussion

Realgar is a class II compound in the biopharmaceutics classification system, characterized by high permeability but poor aqueous solubility^10^,^21^. Improving its dissolution in aqueous solutions is important to increase its bioavailability and thus improve its therapeutic efficacy, as well as to reduce the effective dose for patients. Particle size reduction is a generally accepted strategy to overcome low solubility or dissolution of drugs^37^ on the bases of the Ostwald-Freundlich and Noyes-Whitney equations^38^.

The reasons for the significant improvement in dissolution and bioavailability of e-As_4_S_4_ may include that the particle size of r-As_4_S_4_ was reduced to the nanoscale and the As_4_S_4_ nanoparticles were encapsulated in the water-soluble polymer PVCL-PVAc-PEG in the screwing process, which not only improved the wetting and dispersing properties of As_4_S_4_ nanoparticles but also protected r-As_4_S_4_ from direct oxidation, a major drawback of the milling process^27^. The crucial role of PVCL-PVAc-PEG is to bring a large amount of As_4_S_4_ nanoparticles into aqueous solution rapidly, allowing it to become supersaturated and stable in solution. Such release behavior is important to ensure that As_4_S_4_ nanoparticles move from the stomach to the intestine in a supersaturated state after oral administration, which is beneficial for intestinal absorption and bioavailability^39^.

Although the peak plasma concentrations for e-As_4_S_4_ and r-As_4_S_4_ were reached at almost the same time point, suggesting that the two versions have the same uptake pathway, the peak plasma concentration of e-As_4_S_4_ was much higher than that of r-As_4_S_4_. This was because e-As_4_S_4_ has greatly improved dissolution properties, which promote its uptake by cells in the gastric system. The arsenic concentration in blood for both e-As_4_S_4_ and r-As_4_S_4_ decreased at a constant rate over time, suggesting that the excretion rates of the two were almost the same. As a result, the blood levels of arsenic in mice that received e-As_4_S_4_ remained quite high for 70 h, whereas the levels in mice given r-As_4_S_4_ faded away after 20 h. The longer metabolic half-life in blood should be an advantage of e-As_4_S_4_ in leukemia treatment.

We noted that efficient dosing of As_4_S_4_ is a key factor in obtaining significantly prolonged survival of mice. Because the gastric volume of mice is very limited, the largest available amount for one lavage was 28.8 mg e-As_4_S_4_/1:15–60 suspended in 200 μL saline (containing 1.8 mg As_4_S_4_). When given one lavage per day, mice in the e-As_4_S_4_ group survived longer than those in the r-As_4_S_4_ group; however, this was not statistically significant (Supplementary Fig. S3). This suggested that the concentration of arsenic in blood was not sufficient against the leukemic cells due to the anatomical limitations of the mice. Thus, we gave mice e-As_4_S_4_/1:15–60 or r-As_4_S_4_ twice per day. The mice in the e-As_4_S_4_/1:15–60 group then showed significantly prolonged survival compared to the r-As_4_S_4_ group. We also noticed that e-As_4_S_4_/1:15–60 did not clear leukemia cells completely in the AML mice model to reach disease-free survival (DFS); this was because the treatment regimen that we used differed from that for clinical patients. In clinical treatments, As_4_S_4_ is usually administrated in a consolidation period, with the aim of achieving long-term DFS after obtaining hematological CR by chemotherapy, ATRA treatment, or both^10^. In the present study, e-As_4_S_4_/1:15–60, ATRA, or r-As_4_S_4_ was applied alone in AML mice for the whole treatment period. It is reasonable to expect much better efficacy when e-As_4_S_4_ is used to treat patients in a consolidation period and/or when combined with other therapies.

## Conclusions

In summary, the dissolution rate and bioavailability of r-As_4_S_4_ were both significantly increased by size reduction and polymer encapsulation using the HME technique in a one-step process. The water-soluble e-As_4_S_4_ showed much higher cytotoxicity to HL-60 cells *in vitro* and *in vivo* than r-As_4_S_4_. It also provided relief from AML symptoms and led to longer survival of AML mice. The developed formulation of e-As_4_S_4_ may provide a promising alternative option for AML treatment.

## Materials and Methods

### Materials

Raw As_4_S_4_ (r-As_4_S_4_, CID: 3627253) was purchased from Alfa Aesar Co. (Ward Hill, MA). Polyvinyl caprolactam-polyvinyl acetate-polyethylene glycol (PVCL-PVAc-PEG, Soluplus; average molecular weight = 1.18 × 10^5^ Da) was provided by BASF SE (Ludwigshafen, Germany). All-trans retinoic acid (ATRA) was purchased from Shandong Liangfu Pharmaceutical Co. (Jining, China). Nitric acid and hydrogen peroxide were purchased from Aladdin (ultrapure grade). Other chemicals used in this work were from Beijing Chemical Reagent Company and were of analytical reagent grade.

### Co-extrusion of r-As_4_S_4_ and PVCL-PVAc-PEG

The r-As_4_S_4_ and PVCL-PVAc-PEG at mass ratios of 1:1, 1:9, and 1:15 were fed into a HAAKE MiniLabII co-rotating twin-screw extruder (Thermo Fisher Scientific, Karlsruhe, Germany). The processing conditions were as follows: blending temperature in the mix chamber 120 °C, screw rotation rate 10 rpm, and cycling time 10 min or 60 min. The co-extruded products (e-As_4_S_4_) were further ground in a coffee grinder at room temperature for experiments. The compositions and cycling times for samples are summarized in Table 2.

### Physicochemical characterization of e-As_4_S_4_

#### Solubility in aqueous solutions

Dissolution tests were carried out using the dissolution test apparatus III of the China Pharmacopoeia. Briefly, 20 mg r-As_4_S_4_ or e-As_4_S_4_ containing 20 mg r-As_4_S_4_ was added to a beaker containing 250 mL HCl solution (0.1 M) in a water bath at 37 °C. The stirring rate was 100 rpm. At the designated time points (5, 10, 20, 30, 45, 60, 90, and 120 min after starting), 2 mL supernatant was taken out for measurement and 2 mL fresh medium was added to replenish the solution. The collected samples were completely digested with nitric acid, and then we used ICP-OES to detect arsenic levels (iCAP6300 ICP-OES, Thermo Scientific, USA)^22^. The dissolution rate (*D*) of each sample was calculated according to the following equation^36^:


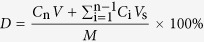


where *C*_i_ is the concentration of As_4_S_4_ at each time point, *V* is the total solution volume in the beaker, *V*_s_ is the sampling volume, n is the total sample number, and *M* is the original total amount of As_4_S_4_.

#### Size distribution measurements

The hydrodynamic diameters of samples including r-As_4_S_4_, e-As_4_S_4_/1:15–60, and washed e-As_4_S_4_/1:15–60 were measured using dynamic light scattering (DLS, Nano ZS90 Zetasizer, Malvern Instruments, Malvern, UK). Briefly, 40 mg e-As_4_S_4_/1:15–60 was added to 3 mL saline, artificial gastric juice, or artificial intestinal juice, followed by sonication in an ultrasound bath for 10 min. For washed e-As_4_S_4_/1:15–60, 40 mg e-As_4_S_4_/1:15–60 was added in 3 mL water, and then thoroughly washed with water, followed by centrifugation (8 × 10^5^ rpm). The precipitate was resuspended in water and immediately measured via DLS. The artificial gastric juice and intestinal juice were prepared according to the standardized formulae in China Pharmacopoeia II.

#### XRD and SEM observations

To prepare samples for X-ray powder diffraction (XRD) and scanning electron microscopy (SEM), e-As_4_S_4_/1:15–60 or r-As_4_S_4_ was added to pure water and sonicated in an ultrasound bath for 10 min. To completely remove the polymer coating, the suspensions were centrifuged (8 × 10^5^ rpm) and the precipitate was rinsed with pure water thoroughly and examined with a D8 Focus powder XRD analyzer (Bruker/AXS, Germany) with a Cu-Kα radiation at 40 kV/40 mA. The diffraction patterns were obtained in a 2*θ* range of 10–90°, using a 0.01° step size and 26 s step time.

For SEM examination, the cleaned precipitate was resuspended in water and dropped onto silicon wafers. After drying in air, the sample was coated with gold-palladium for 30 s under an argon atmosphere using an SCD 500 high-vacuum sputter coater (Leica Microsystems GmbH, Germany). The SEM examination was performed using a Quanta 200F environmental scanning electron microscope (FEI Company, Hillsboro, OR, USA), operated at an accelerating voltage of 15 kV. Diameters of up to 150 particles were measured using the Nano Measurer 1.2.5 program (Fudan University, China) and the average diameter was calculated.

### Preparation of suspension samples of r-As_4_S_4_ or e-As_4_S_4_

To obtain reproducible data, the largest possible suspension samples of r-As_4_S_4_ or e-As_4_S_4_ for *in vivo* and *in vitro* experiments were prepared according to the same standard process. Briefly, 2.5 mg r-As_4_S_4_ or 40 mg e-As_4_S_4_/1:15–60 (containing 2.5 mg r-As_4_S_4_) was added to 3 mL saline and sonicated in an ultrasound bath for 10 min at room temperature. The suspension was freshly prepared prior to the experiments.

### Bioavailability assay

Eight male Sprague Dawley (SD) rats of 200 g (Vital River Laboratory Animal Technology Co. Ltd., Beijing) were divided randomly and equally into two groups. A single dose of r-As_4_S_4_ or e-As_4_S_4_/1:15–60 was given (45 mg As_4_S_4_/kg) by intragastric administration. A blood sample of 200 μL was collected from the retro-orbital plexus at the following time points post administration: 0.5, 1, 2, 4, 8, 12, 24, 36, 48, and 72 h. Samples were transferred to 25-mL conical flasks, to which 3 mL nitric acid and 1 mL hydrogen peroxide were added. Next, the conical flasks were heated in a boiling water bath for at least 2 h until the liquid volume was reduced to 1 mL. The samples were cooled to room temperature and diluted to 4 mL with 2% nitric acid, and then we measured arsenic using hydride generation atomic fluorescence spectrometry (AFS-8230 HG-AFS, Beijing Titan Instruments Co., Ltd., Beijing, China) according to a reported procedure^40^.

### Cell culture

HL-60 cells (an acute promyelocytic leukemia cell line) were purchased from the Cell Resource Center of the Chinese Academy of Medical Sciences (Beijing, China) and cultured in RPMI 1640 medium (Hyclone, Thermo Scientific) supplemented with 10% fetal bovine serum (FBS, Gibco Life Technologies), 100 U/mL penicillin, and 100 U/mL streptomycin in a humidified atmosphere of 5% CO_2_ at 37 °C.

### Cell viability assay

Cell viability was measured using a cell-counting kit (CCK-8, Dojindo, Japan) according to the manufacturer’s protocol. To establish a standard curve for the quantification of cell numbers, HL-60 cells were seeded in 96-well U-bottom plates at densities of 2, 4, 8, 16, 32, and 64 × 10^4^ cells/well and 20 μL CCK-8 reagent was added to each well. After incubation at 37 °C for 1 h, the absorbance was read at 450 nm with a Synergy H1 microplate spectrophotometer (BioTek Instruments, Winooski, VT). Next, HL-60 cells were seeded into the plates at a density of 8 × 10^4^ cells per well. After incubation with r-As_4_S_4_, e-As_4_S_4_/1:3–60, e-As_4_S_4_/1:9–60, or e-As_4_S_4_/1:15–60 at 10, 20, and 40 mg/L of As_4_S_4_ or with PVCL-PVAc-PEG at 150, 300, and 600 mg/L for 3–72 h at 37 °C, 20 μL CCK-8 reagent was added to each well and incubated at 37 °C for 1 h. The absorbance value was read at 450 nm and transformed into the number of viable cells using the standard curve. Measurements were carried out in quadruplicate.

### Cell apoptosis assay

Flow cytometric analysis of HL-60 cells double-stained with apoptosis annexin V-FITC/PI (eBioscience, Vienna, Austria) was used to determine cell apoptosis. Briefly, HL-60 cells were seeded in 24-well plates and incubated with e-As_4_S_4_/1:15–60 at different concentrations of As_4_S_4_ for 6–48 h. After centrifugation and washing with PBS, cells were resuspended in binding buffer. Then, annexin V-FITC and PI were added, followed by a 10-min incubation at room temperature, and were analyzed using a flow cytometer (Accuri C6; BD Company, Franklin Lakes, NJ). In total, 1.5 × 10^4^ cells were measured and the data acquired were analyzed with the CFlow Plus software.

Apoptotic cells were also examined by microscopy following Hoechst 33342 (Beyotime Biotechnology, Haimen, China) staining. Cells were seeded in 24-well plates and a suspension of e-As_4_S_4_/1:15–60 at 40 mg/L of As_4_S_4_ was added. After 48 h incubation, the cells were harvested in a 1.5-mL concentration tube, washed twice with PBS, and suspended in 1 mL medium. Hoechst 33342 at 5 μL/mL was added to the tube and incubated for 25 min at 4 °C in the dark. Then the cells were washed twice with PBS to remove unbound dye and imaged with a fluorescence microscope (IX71, Olympus, Tokyo, Japan).

### AML animal model and drug administration

Five-week old female NOD/SCID mice were maintained in the Experimental Animal Center of the Institute of Basic Medical Sciences, Chinese Academy of Medical Sciences (Beijing, China) under specific-pathogen-free conditions. All animal experiments were conducted in accordance with the approved guidelines and were approved by the committee on Animal Care and Use of the Institute of Basic Medical Sciences, Chinese Academy of Medical Sciences & Peking Union Medical College. Animals were acclimatized to laboratory conditions for 1 week prior to the experiments. HL-60 cells (1 × 10^6^), suspended in 100 μL EDTA/PBS, were injected intravenously into sublethally irradiated (250 cGy) NOD/SCID mice. At about 20 days after transplantation, the mice showed signs of leukemia.

On day 20 after HL60 cell transplantation, the mice were randomly divided into four groups (*n* = 13) as follows: e-As_4_S_4_, r-As_4_S_4_, ATRA, and saline. Given that the gastric volume of mice is very limited, mice in the e-As_4_S_4_ and r-As_4_S_4_ groups were lavaged twice per day, each time with e-As_4_S_4_/1:15–60 of 28.8 mg or r-As_4_S_4_ of 1.8 mg suspended in 200 μL saline per mouse. For mice in the ATRA group, 0.18 mg ATRA in 200 μL saline was given once per day. For mice in the saline group (control), 200 μL saline was administrated twice per day. After 3 weeks of treatment (on day 41 after cell transplantation), five mice were randomly selected from each group and sacrificed. The spleen, peripheral blood, and bone marrow were collected for the following examinations. The other eight mice left in each group continued to receive treatment until death. During the treatment, the body weight of each mouse was measured every other day.

### Flow cytometry to examine CD33^+^ cells in blood and bone marrow

The peripheral blood and bone marrow from femur flushing were collected and erythrocytes were excluded using blood cell lysis buffer (Beckman Coulter, Krefeld, Germany). PE anti-human CD33 antibody (BioLegend, San Diego, CA) was used to identify HL-60 cells via flow cytometry (C6 Accuri flow cytometer, BD Company, Franklin Lakes, NJ) according to the manufacturer’s protocol.

### Histology and immunohistochemistry

Spleen and liver tissues collected from mice receiving the different treatments for 3 weeks were fixed in 10% formalin over 24 h, followed by embedding in paraffin wax, and then were cut into 4-μm sections and stained with H&E. The sections were also stained with anti-human CD33 (1:100 dilution, BioLegend, San Diego, CA). To enhance immunostaining, antigen retrieval was performed by heating in citrate buffer using a microwave oven. The rest of the immunohistochemical staining followed a reported procedure^41^. Slides were counterstained with Mayer’s hematoxylin and photographed with an optical microscope (Olympus BX53; Tokyo, Japan).

### Statistical Analysis

Student’s *t*-tests (two-tailed) were used to assess the statistical significance of experimental results, except the survival data. Survival was assessed using the log-rank test (Mantel-Cox). All statistical analyses were performed using SAS software (ver. 8.2; SAS Institute. *P < 0.05 and **P < 0.01).

## Additional Information

**How to cite this article**: Ma, Q. *et al*. Fabrication of water-soluble polymer-encapsulated As_4_S_4_ to increase oral bioavailability and chemotherapeutic efficacy in AML mice. *Sci. Rep*. **6**, 29348; doi: 10.1038/srep29348 (2016).

## Supplementary Material

Supplementary Information

## Figures and Tables

**Table 1:** AUC of single oral administration for e-As_4_S_4_ and r-As_4_S_4_.

Formulation	r-As_4_S_4_	e-As_4_S_4_/1:15–60
AUC_0-72_ (mg h/L)	12.5 ± 5.2	158.5 ± 17.5**

Notes: **P < 0.01 versus r-As_4_S_4_ by *t*-test. (n = 4, mean ± SD).

**Table 2:** Composition and cycling time of e-As_4_S_4_.

	r-As_4_S_4_ (g)	PVCL-PVAc-PEG (g)	Cycling time (min)
e-As_4_S_4_/1:1–10	4	4	10
e-As_4_S_4_/1:9–10	4	36	10
e-As_4_S_4_/1:15–10	4	60	10
e-As_4_S_4_/1:15–60	4	60	60

The English in this document has been checked by at least two professional editors, both native speakers of English. For a certificate, please see: http://www.textcheck.com/certificate/5IoxPF.
