# Supplementary material for: Fabrication of water-soluble polymer-encapsulated As4S4 to increase oral bioavailability and chemotherapeutic efficacy in AML mice
Source: Sci Rep. 2016 Jul 7;6:29348. doi: 10.1038/srep29348 (PMC4935940; doi:10.1038/srep29348)
Supplement: Supplementary Information [file srep29348-s1.pdf]

Fabrication of water-soluble polymer encapsulated-As_4S_4 to increase the oral bioavailability and chemotherapeutic efficacy in AML mice

Qiang Ma[#], Chuan Wang[#], Xiaojin Li, Hua Guo, JieMeng, Jian Liu[*] & Haiyan Xu[*]

Institute of Basic Medical Sciences Chinese Academy of Medical Sciences, School of Basic Medicine Peking Union Medical College, Beijing 100005, P. R. China

[#] These authors contributed equally to this work

[*] Correspondence: xuhy@pumc.edu.cn, liujian@ibms.pumc.edu.cn

Supplementary Figure S1.

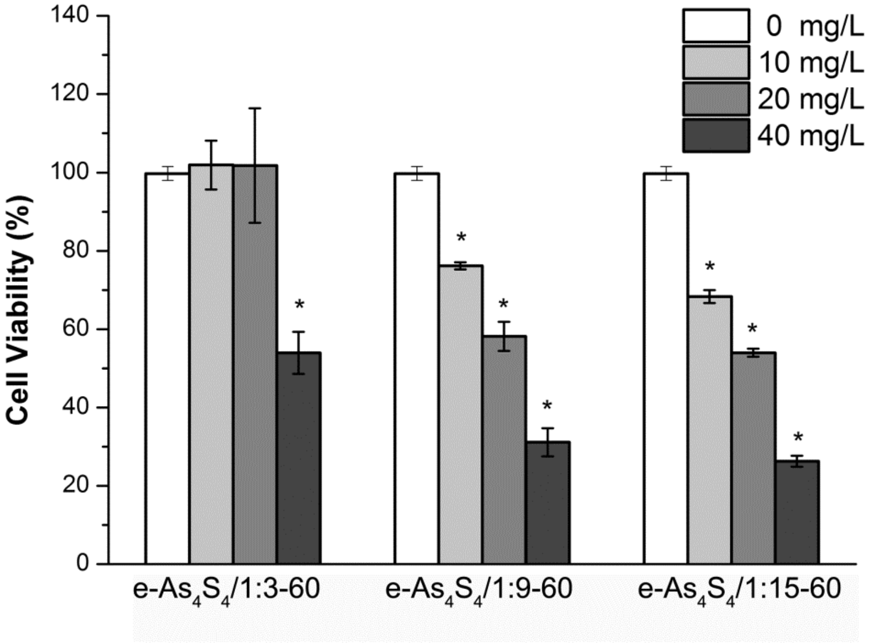

Supplementary Figure S1. Cytotoxicity of e-As_4S_4 on HL60 cells. HL60 cells were treated with saline, e-As_4S_4/1:3-60, e-As_4S_4/1:9-60 and e-As_4S_4/1:15-60 (n=4) for 48 h. Student's *t*-test (two-tailed) was performed to assess statistical significance of experimental results vs saline (* $P<0.05$).

Supplementary Figure S2.

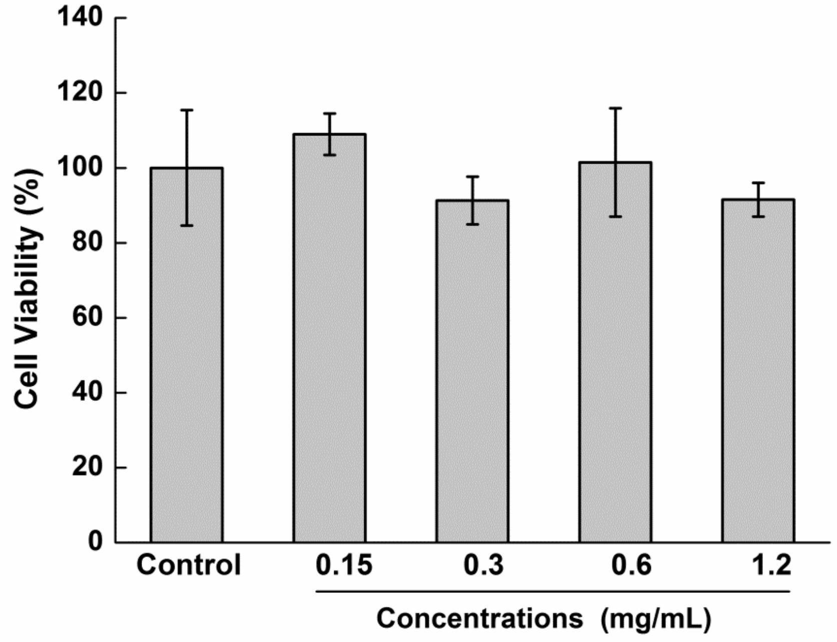

Figure S2. Cytotoxicity of PVCL-PVAc-PEG (Soluplus®) on HL60 cells. HL60 cells were treated with **PVCL-PVAc-PEG** at different concentration for 72h. Student's *t*-test (two-tailed) was performed to assess statistical significance of experimental results vs saline (* $P<0.05$).

Supplementary Figure S3.

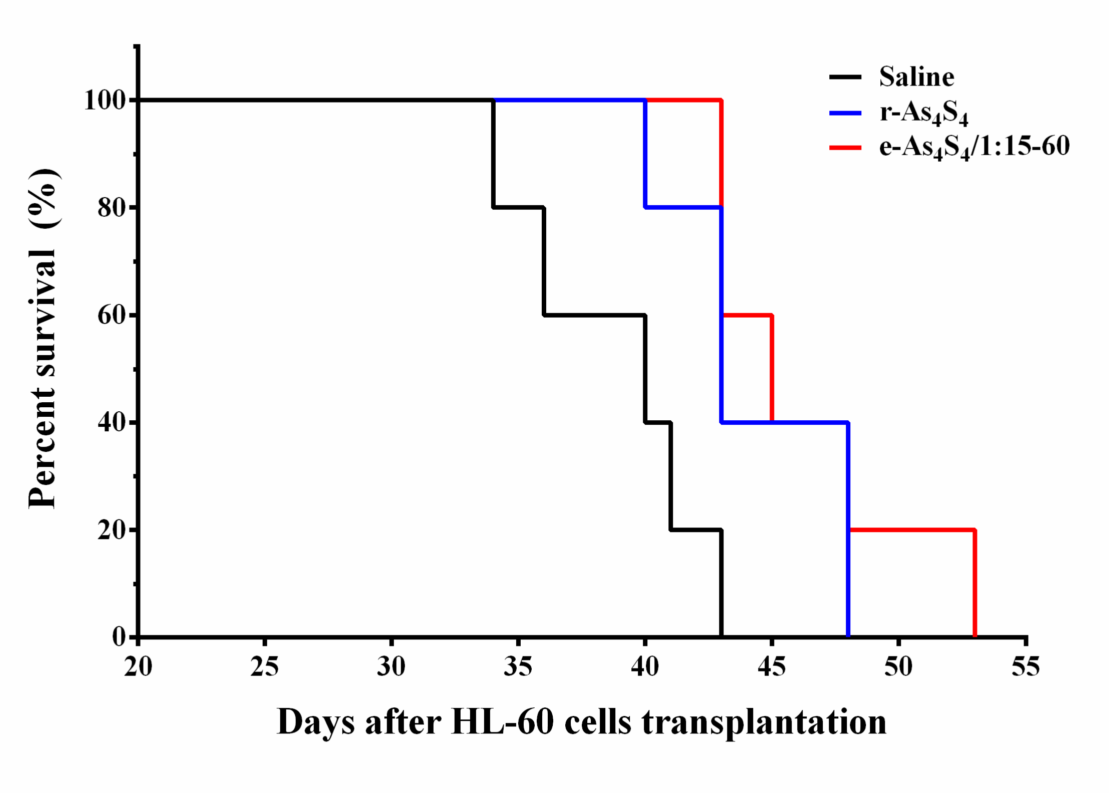

Supplementary Figure S3. ***In vivo*** **therapeutic efficacies of the half-dose r-As_4S_4, e-As_4S_4 and ATRA.** Survival curve of AML mice treated with saline (blue line), r-As_4S_4 (red line) or e-As_4S_4/1:15-60 (green line), n=5, Log-rank test (Mantel-Cox) was used to assess statistical significance: e-As_4S_4 vs saline (P<0.0078), r-As_4S_4 vs saline (P=0.042), e-As_4S_4 vs r-As_4S_4 (P=0.4803). NOD/SCID mice were following intravenously injected with HL-60 cells (1×10^6 cells per mouse) after 250 cGy γ-irradiation. After 20 days, the treatment was started with intragastric administration of saline, r-As_4S_4 (1.8 mg per mouse per day) or e-As_4S_4/1:15-60 (28.8 mg per mouse per day).
